# Is it possible to encourage TB testing and detect missing TB cases via community-level promotion of a self-screening mobile application? Quasi-experimental evidence from South Africa

**DOI:** 10.1136/bmjhci-2024-101179

**Published:** 2025-05-31

**Authors:** Kate Rich, Ronelle Burger, Deanne Goldberg, Harry Moultrie, Matthias Rieger

**Affiliations:** 1 Department of Economics, Stellenbosch University, Stellenbosch, South Africa; 2 School of Economics and Finance, University of the Witwatersrand, Johannesburg, South Africa; 3 Clinton Health Access Initiative, Johannesburg, South Africa; 4 Centre for Tuberculosis, National Institute for Communicable Diseases, Division of the National Health Laboratory Service, Johannesburg, South Africa; 5 Erasmus School of Health Policy and Management, Erasmus Universiteit Rotterdam, Rotterdam, The Netherlands; 6 International Institute of Social Studies, Erasmus Universiteit Rotterdam, The Hague, The Netherlands

**Keywords:** Infectious Disease Medicine, Delivery of Health Care, Global Health, Program Evaluation, Smartphone

## Abstract

**Objectives:**

While mobile health (mHealth) interventions are widespread, few studies assess impacts at the population level in low-income and middle-income countries. South Africa’s tuberculosis (TB) burden is high, and a substantial share of cases remain undiagnosed. We evaluate the impacts of community activations of TBCheck—a WhatsApp/USSD-based chatbot that allows individuals to evaluate themselves for TB risk.

**Methods:**

We use a quasi-experimental approach comparing treated and control subdistricts nationally before and after community activations using dashboard data from the TBCheck platform and weekly or quarterly subdistrict TB test data from the National Health Laboratory Service. Dependent variables are the number of self-screening tests on the platform, total tests and number of positive tests per subdistrict. We employ dynamic difference-in-difference models accounting for subdistrict unobservables and time trends using weekly data, and synthetic control methods matching on preintervention trends in outcomes using quarterly data.

**Results:**

Impact estimates suggest an increase in the number of self-screening tests on the platform (487.53, p-value<0.01) as well as TB tests (107.90, p-value=0.05) in treated relative to control subdistricts due to intervention activities in the week of the intervention. After 2 weeks, impacts on the number of self-screening tests are insignificant (−6.18, p=0.23), and after 1 week, impacts on TB tests are insignificant (36.44, p-value=0.32).

**Discussion and conclusion:**

Activation activities associated with TBCheck led to short-lived and variable impacts on uptake and tests in target subdistricts. Alternative strategies are required for sustained uptake of such mHealth tools.

**WHAT IS ALREADY KNOWN ON THIS TOPIC**

- Mobile health (mHealth) applications for infectious disease self-screening have been widely deployed, in particular since COVID-19. However, counterfactual evidence at the population level on the uptake and effectiveness of such self-screening apps remains very limited in low-income and middle-income countries.

**WHAT THIS STUDY ADDS**

- This study adds the first population-level and clinical outcome evidence exploiting counterfactual methods on a major mHealth intervention to find ‘missing’ tuberculosis cases in the sub-Saharan African context.

**HOW THIS STUDY MIGHT AFFECT RESEARCH PRACTICE OR POLICY**

- While mHealth interventions for disease screening are easily scalable from a technical point of view, this study shows that sustained and wide-scale uptake remains an important barrier to effectiveness.

## Introduction

 Promotion of self-screening and early detection of communicable diseases is a priority for public health yet challenging due to behavioural complexity. Mobile health (mHealth) has been identified as an important solution. The term broadly collects mobile wireless technologies for public health, ranging from the basic provision of information and services through SMSes to more sophisticated smartphone applications.^1^ MHealth interventions have been used to promote screening and testing for COVID-19, TB and Ebola in African countries.^2^ Despite widespread application, little is known about the impacts of such applications on case identification at the population level. A recent systematic review of mHealth platforms for case identification in African countries reports that assessments tend to focus on the number of self-screen tests via mHealth platforms and do not consider the translation of positive self-screens to testing and case identification.^2^

This study contributes an in-depth, rigorous assessment of the TBCheck platform’s impact on self-administered screening for potential TB, but also on testing and case identification. TBCheck was developed to provide scalable, affordable support for post-COVID TB case detection in South Africa (SA). The country accounts for 3.6% of the global TB burden and is among the 14 countries with the highest burden of TB, TB/HIV and multidrug resistant TB.^3^ TB-related mortality is the leading cause of death in SA.^4 5^ The high rate of HIV coinfection continues to amplify both the spread and the mortality of the epidemic.^6^ SA’s 2018 TB prevalence survey found 852 bacteriologically confirmed pulmonary TB cases per 100 000 and a total of 150 000 ‘missing’ ones where people tested positive for TB in the survey but had not yet been tested or diagnosed and were therefore also not on treatment.^7 8^ Treatment delays are associated with a higher likelihood of hospital referral and mortality.^9^

The TBCheck platform was conceived as a response to ‘missing’ TB cases, as a way for people to screen themselves for possible infection and encourage those identified as high risk to get tested for TB. The tool was also envisaged as a response to the dramatic decline in TB testing during the pandemic. TBCheck followed the design of the HealthCheck COVID-19 self-assessment tool, which screened over 1 million people.^10^ The HealthCheck tool’s COVID self-screen tests were achieved when these were mandated for entry to government buildings to study and work, so it remains unclear if similar uptake will occur for TB where screening is not compulsory. Similar to HealthCheck, TBCheck is accessed through the National Department of Health’s WhatsApp and USSD chatbot support service. The TBCheck chatbot asked users five questions on their symptoms and risk of TB, and then classified users’ risk and, if indicated, advised them to test for TB. TBCheck was available nationally in March 2021 with relatively low take up, but in late 2022 and early 2023, week-long activation campaigns were undertaken in communities and clinics in eight subdistricts to promote awareness and use of the tool for self-screening.

This study aimed to assess whether these community activations promoting a mobile health TB screening platform could increase uptake of self-screening, as well as the number of TB tests and positive cases identified, and whether any increases would be sustained over time. The emphasis was on interventions that would be replicable and sustainable within the constraints of the public health system and budgets.

## Method

### Mobile health platform and interventions

TBCheck was created for the South African Department of Health, and the research team did not influence the screening questions and the overall design of the platform. The screening questions were based on the standard WHO four symptom screening, whose quality and reliability may depend on context and clinical conditions.^11^ The screening procedure on the platform also reflects high-risk groups identified under the Targeted Universal TB Testing approach approved by SA authorities, including the Western Cape. These cover individuals living with HIV/AIDS, individuals with prior TB infection and individuals with household TB contacts (see details in online supplemental material A).

The study assesses the promotion of the TBCheck platform via community activation (interventions) in purposively selected rural and urban subdistricts (see details in online supplemental material B regarding clinic numbers and ease of access to make the interventions feasible and cost-effective). Activities lasted a week in each subdistrict and reached specific populations (see details in online supplemental material C1). Promoters were assigned to public clinics, shopping centres and taxi ranks, with roughly 3–6 promoters selected from the community at each site. Promoters told individuals about TBCheck and encouraged screening via own phones. Promoters were available to assist users who experienced challenges with completing the screening. Posters promoting TBCheck, including the phone numbers used to access it, were put up in clinics and selected areas in the intervention subdistricts. Additionally, in three subdistricts, small incentives were offered: in Big Five Hlabisa, clinic staff were offered breakfast packs and clinic attendees were offered snacks; in Nelson Mandela Bay subdistrict A and Johannesburg subdistrict D, customers in selected shopping centres were offered a chance to win branded merchandise for screening completion.

### Data

The main outcomes were the number of self-screenings using TBCheck, total TB tests and number of positive TB tests per subdistrict per week. As an additional outcome, we also considered the weekly positivity rate (positive tests divided by total tests). The number of self-screening tests (henceforth ‘screens’ for short) and positive screens (screens resulting in a recommendation to get tested; a ‘positive’ or presumptive screening result does not necessarily mean an individual would test positive for TB) were obtained from usage data provided by the TBCheck developer, Reach Digital Health. Screens were assigned to subdistricts based on users’ self-reported address by Reach Digital Health using Google Location Services.

Data on individual TB tests in each subdistrict were obtained from the National Health Laboratory Service (NHLS). We limited the analysis to 2022 and 2023 to avoid picking up spurious testing trends over a longer period. Only GeneXpert tests were included (a rapid, molecular TB test available nationally since 2011). Population numbers come from the District Health Barometer 2022/23.

### Statistical model

Our approach is quasi-experimental as a randomised trial was infeasible; we constructed a counterfactual that followed similar trends (netting out time-invariant baseline differences) in the main outcome variables of interest prior to the interventions.

We estimated dynamic difference-in-difference models (panel event study models) as our primary analysis, which allowed us to explore dynamics in any effects of the interventions. Difference-in-difference models assume that intervention and control subdistricts would have followed similar trends in the absence of the intervention (the parallel trend assumption). Dynamic difference-in-difference models include leads and lags of the intervention indicator. Leads are indicators for weeks before the intervention, while lags are indicators for weeks after the intervention. The leads show the average difference between treated and control subdistricts in each week before the intervention, allowing us to assess whether the assumption of parallel trends held in the weeks before the intervention. If treated and control subdistricts followed similar trends before the intervention, we would see no significant difference in the average number of screens between treated and control subdistricts in the weeks leading up to the intervention. The lags show the average difference between treated and control subdistricts in the weeks following the intervention. We included 8 weeks before and after the intervention, with the eighth lead and lag, respectively, representing the accumulated effect in all periods more than 8 weeks before and after the intervention. Dynamic difference-in-difference models group the data according to the number of weeks before the start of the intervention for each group. This is appropriate for a staggered intervention roll-out such as the one in this study. We estimated this dynamic model in STATA V.18.0 using *eventdd*.^12^ This analysis covered the period 2022 week 1 to 2023 week 8 to avoid confounding by other promotional campaigns not evaluated here beginning in 2023 week 9.

We also estimated the mean effect of the interventions over the whole postintervention period on TBCheck screens and positive screens and on total TB tests and positive tests using fixed-effects models with subdistrict and week-year fixed effects. The subdistrict fixed effects net out any confounders that do not vary over the analysis period. The temporal fixed effects account for nationwide seasonalities and shocks such as changes in the country’s TB strategy or COVID-19 policies. Clustered SEs allow for arbitrary correlation of observations within subdistricts over time. These models obscure any variation in effects over time; this is thus considered a secondary analysis.

Interventions may have had significant impacts in some subdistricts but not others. As another secondary analysis, we also planned the use of the synthetic control method on quarterly data to attempt to test for sustained and heterogeneous effects for each subdistrict individually. This method is useful when studying intensive interventions at the population level that are not randomised, when outcomes are based on aggregate data, and when treated units are few.^13^ For each treated subdistrict, the estimator searches for a weighted average of control subdistricts that followed similar trends to the treated subdistricts prior to the intervention period (the synthetic counterfactual). We rely on quarterly data which are relatively smoother than weekly data to be able to match on preintervention trends. A limitation of the synthetic control approach is that highly variable data (such as weekly frequency data) cannot be analysed, in particular when impact estimates are very small.^14^ While the previous analyses using weekly data covered the period from 2022 week 1 to 2023 week 8, here the analysis covered the period 2021 quarter 4 to 2023 quarter 1 to allow us to match on at least four quarters before the beginning of the interventions. Two subdistricts were excluded from the pool of synthetic controls because they were the site of a promotional campaign in schools that was associated with large increases in screens beginning in 2023 week 9. To gauge the precision of estimates, we relied on pseudo p-values. We estimated the model using *synth* and *synth_runner*.^15^

## Results

### Descriptive statistics

We excluded screens where the subdistrict was missing because the user had not reported an address. A total of 3104 screens, or 9.59% of the total of 32 354 screens over the period we consider (2022 week 1 to 2023 week 8), were excluded.

Table 1 (panel A) shows that in the preintervention period (2022 weeks 1–39), intervention subdistricts averaged per week 4.25 screens (SD=30.61), 1.81 positive screens (SD=11.75), as well as 516.06 TB tests (SD=278.55) and 34.54 positive TB tests (SD=25.94). They thus exceeded control districts averaging per week 1.39 screens (SD=6.33), 0.6 positive screens (SD=3.12), as well as 189.49 TB tests (SD=348.53) and 13.65 positive TB tests (SD=23.10).

**Table 1:** Descriptive statistics for intervention and control subdistricts before interventions (panel A) (2022 weeks 1–39) and after first intervention start (panel B) (2022 week 40–2023 week 8), and average population totals across subdistricts in 2021 (panel C)

	Total				Control	Intervention	P valuediff.
	N	Weekly mean	Min	Max	Weekly mean	Weekly mean	
*Panel A*: preintervention period							
Number of total screens (dashboard)	9243	1.48 (8.40)	0	505	1.39 (6.33)	4.25 (30.61)	<0.01
Number of positive screens (dashboard)	9243	0.64 (3.75)	0	189	0.60 (3.12)	1.81 (11.75)	<0.01
Number of tests (NHLS)	9594	200.10 (351.27)	0	6243	189.48 (348.53)	516.06 (278.55)	<0.01
Number of positive tests (NHLS)	9594	14.33 (23.49)	0	378	13.65 (23.10)	34.54 (25.94)	<0.01
*Panel B*: postintervention period							
Number of total screens (dashboard)	4977	3.13 (26.82)	0	918	2.20 (12.70)	29.57 (126.76)	<0.01
Number of positive screens (dashboard)	4977	1.42 (10.06)	0	363	1.07 (5.53)	11.28 (45.08)	<0.01
Number of tests (NHLS)	5166	189.68 (317.10)	0	5334	179.74 (314.35)	485.41 (249.02)	<0.01
Number of positive tests (NHLS)	5166	14.52 (23.87)	0	357	13.82 (23.40)	35.23 (28.13)	<0.01
*Panel C:* Year 2021	**No of subdistricts**	**Mean**	**Min**	**Max**	**Mean**	**Mean**	
Population of subdistricts	241	234 795.60	8992	1 529 355	226 335.60	481 192.60	<0.01

Notes: SD in brackets. Sample sizes (N) in panels A and B are derived from week x subdistrict observations; 237(246) subdistricts in dashboard (NHLS) variables. Panel C shows average population in 2021 across 241 subdistricts. Discrepancies across panels in the number of subdistricts are due to slightly different data coverage and aggregation (e.g., NHLS data divides Buffalo City into several subdistricts, while the population data lumps them into a single unit).

NHLS, National Health Laboratory Service.

These baseline differences in means were statistically significant (p≤0.01). These differences magnified in the postintervention period (panel B): for example, intervention subdistricts had 2.86 more screens preintervention and 27.30 more screens postintervention, which equates to a ‘difference-in-difference’ of 24.44 screens. Online supplemental material C2 details corresponding large increases in screens between the preintervention and postintervention period for each intervention subdistrict individually. Online supplemental material C3C3 shows trends in screens for intervention areas and the mean across control subdistricts. With the launch of the intervention activities, there were large but brief spikes in intervention subdistricts.

Intervention subdistricts had larger population counts on average (481 192.6) than control subdistricts (226 335.6) (p-value<0.01). The implied average weekly TB tests per 100 000 were thus 107.23 in intervention and 83.72 in control subdistricts, hinting at the extent of the TB burden. Extrapolating using the national prevalence^8^ applied to the average intervention subdistrict implies 4099.76 TB cases (852×(481 192.6/100 000)).

### Dynamic difference-in-difference models

**Figure 1:**
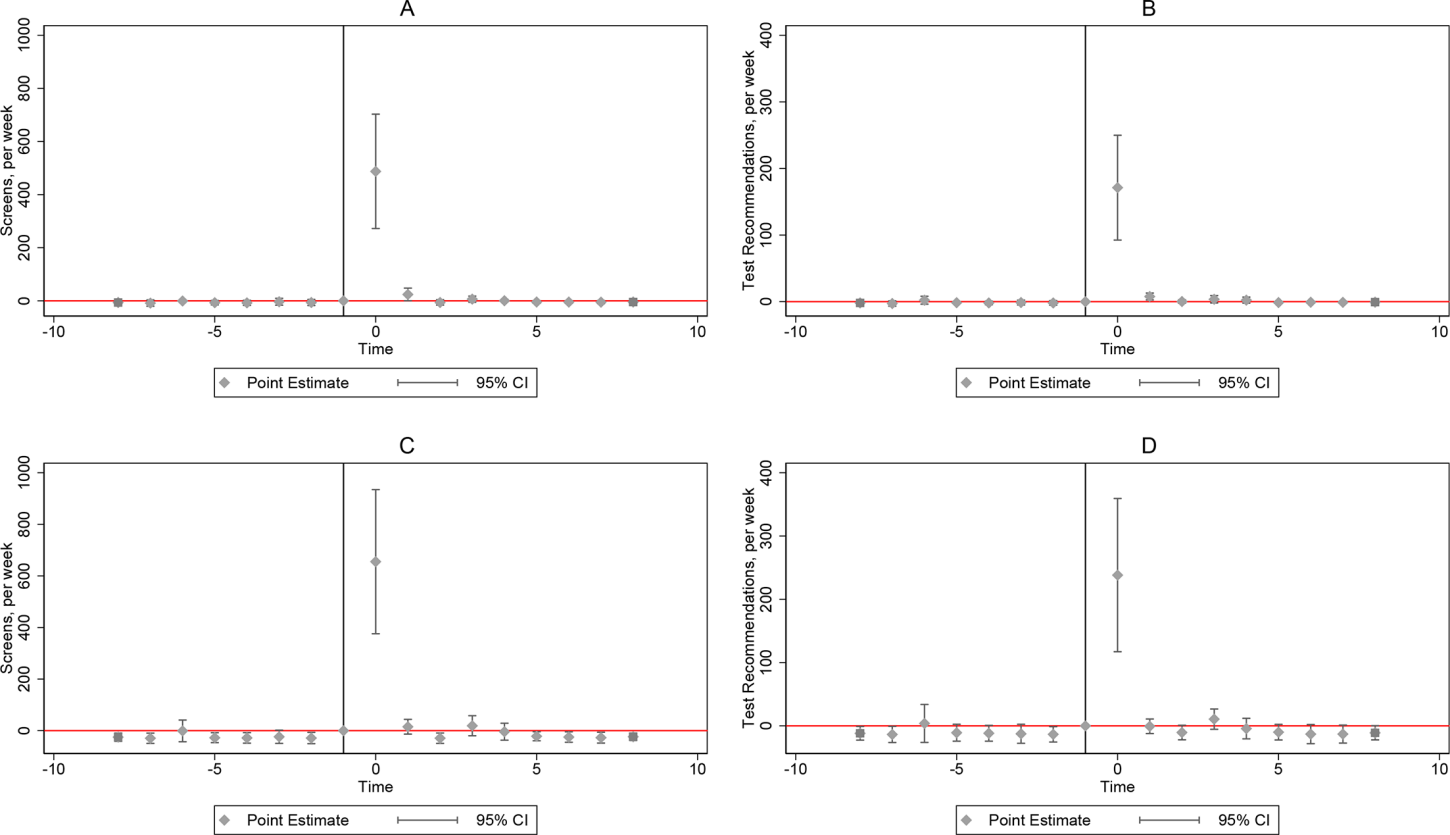
Dynamic impacts of intervention on total TBCheck screens and positive screens, dynamic difference-in-difference models using weekly TBCheck usage data (platform’s dashboard), 2022 week 1–2023 week 8. Note: 95% confidence bands are shown. Panels A and C show impacts on the number of screens, while panels B and D show impacts on the number of positive screens (screens resulting in a recommendation to get tested). Panels A and B show the results of the subdistrict-level analysis, while panels C and D show a district-level analysis comparing treated and untreated districts to account for possible spillovers to untreated subdistricts within the same district as described in-text. A district is considered treated if one of its subdistricts was treated.

**Figure 2:**
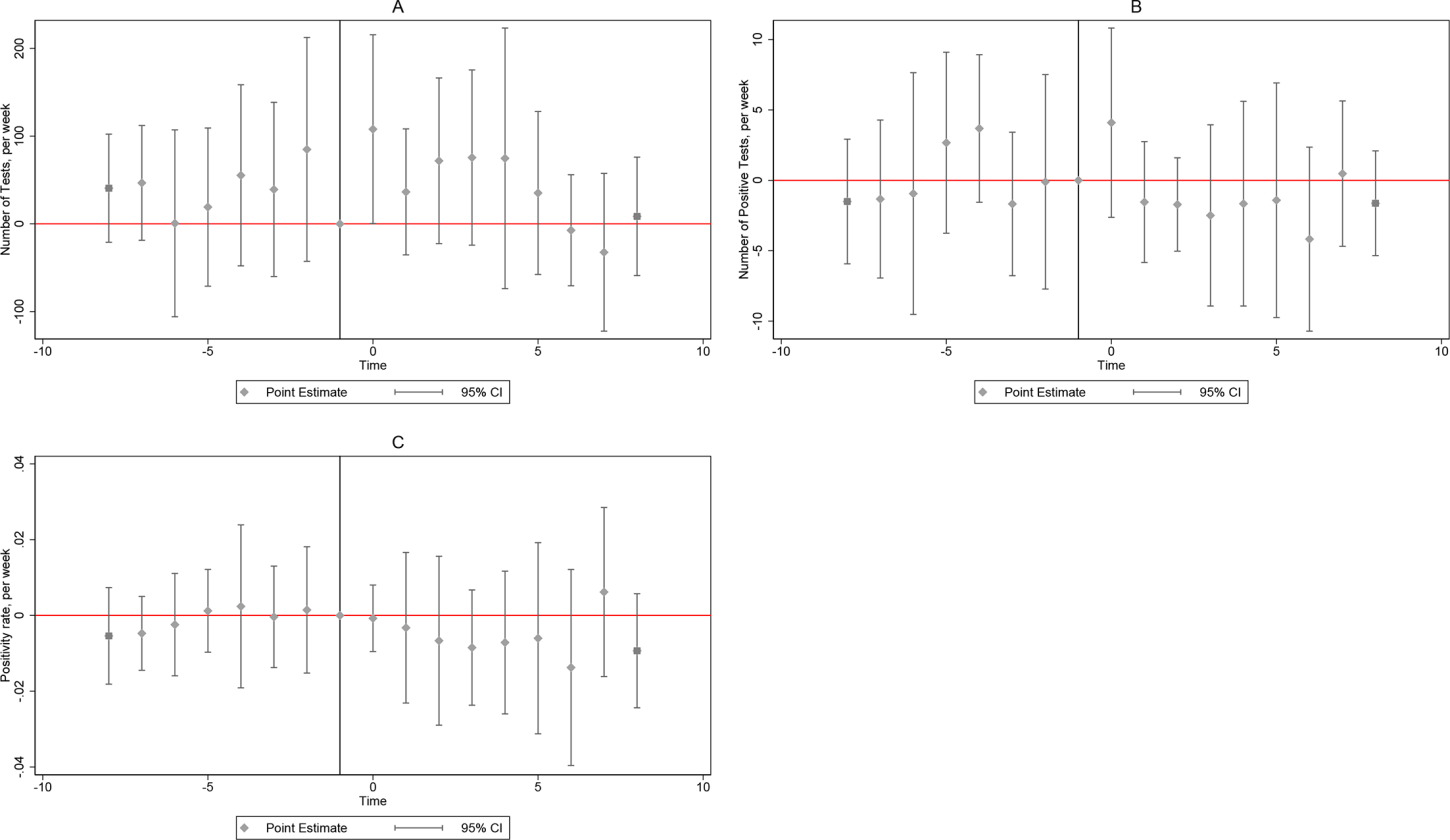
Dynamic impacts of intervention on total TB tests (panel A), positive TB tests (panel B) and positivity rate (panel C), dynamic difference-in-difference models using weekly NHLS data, 2022 week 1–2023 week 8. Note: 95% confidence bands. NHLS, National Health Laboratory Service.

Our primary results are presented in figure 1 (screens, TBCheck dashboard) and figure 2 (tests, NHLS). Prior to the intervention activities, there were no significant and qualitatively important differences in the number of screens in intervention and control subdistricts, supporting the parallel trends assumption and the credibility of our strategy. With the start of intervention activities, screens increased by 487.53 (p-value<0.01), but quickly fell to 23.99 (p-value=0.05) in the following week, and effects vanished or diminished qualitatively thereafter (figure 1 panel A). Similarly, figure 1 panel B shows an initial increase in the number of positive screens by 171.22 (p-value≤0.01) relative to control subdistricts, followed by a rapid decline. Figure 2 panel A shows a significant increase in tests only in the activation week (107.90, p-value=0.05). There are no significant effects on positive tests (panel B) nor the positivity rate (panel C) throughout.

### Fixed effects models

Table 2 panel A shows that there were also significant effects on the mean number of screens over the whole postintervention period. These fixed-effects models net out time-invariant baseline differences between intervention and control. In the preferred specification with subdistrict and week-year fixed effects (column 4), intervention activities were associated with an increase of 36.49 (p-value=0.001) screens relative to control subdistricts, averaged over the whole postintervention period. Table 2 panel B shows that the number of tests per week did not increase significantly following intervention activities when averaged over the whole postintervention period: the preferred model with subdistrict and time fixed effects (column 4) suggests a non-significant decrease of −11.66 tests (p-value=0.52) (a robustness check using log-transformed dependent variables showed non-significant and small increases too; online supplemental material C4).

**Table 2:** Average impact of the intervention over the whole postintervention period, weekly data

	(1)	(2)	(3)	(4)	(5)
*Panel A*					
Impact	34.81*** (10.62)	34.52*** (10.70)	36.85*** (11.16)	36.49*** (11.27)	36.83*** (11.28)
Week FEs		x		x	x
Subdistrict FEs			x	x	x
R^2^	0.04	0.05	0.07	0.08	0.08
N	14 220	14 220	14 220	14 220	12 120
*Panel B*					
Impact	254.60*** (76.66)	271.66*** (77.94)	−28.11 (18.40)	−11.66 (17.92)	−10.58 (18.14)
Week FEs		x		x	x
subdistrict FEs			x	x	x
R^2^	0.01	0.01	0.93	0.94	0.94
N	14 760	14 760	14 760	14 760	12 660

Panel A: average impact of intervention on total number of TBCheck screens (dashboard), fixed-effects models using weekly TBCheck usage data, 2022 week 1–2023 week 8. Panel B: average impact of intervention on total number of TB tests (NHLS), fixed-effects models using weekly NHLS data 2022, week 1–2023 week 8.

Notes: point estimates denoted ‘impact’ capture the average impact of the intervention on self-screens via TBCheck (panel A) and TB tests (panel B) compared with control districts. FE denotes fixed effect (exploiting within subdistrict and week variation by adding dummies); ‘x’ indicates whether week and/or subdistrict fixed effects were included. Column 1 includes no fixed effects, column 2 includes week fixed effects, column 3 includes subdistrict fixed effects, columns 4 and 5 include both week and subdistrict fixed effects. Column 4 is the preferred model due to the full inclusion of fixed effects. Furthermore, column 5 (a sensitivity check with respect to column 4) excludes subdistricts in districts with treated subdistricts to account for possible spillovers. SEs in parentheses are clustered to account for arbitrary error correlation at the subdistrict level.

*P<0.1, **p<0.05, ***p<0.01.

NHLS, National Health Laboratory Service.

### Synthetic control estimates

**Figure 3:**
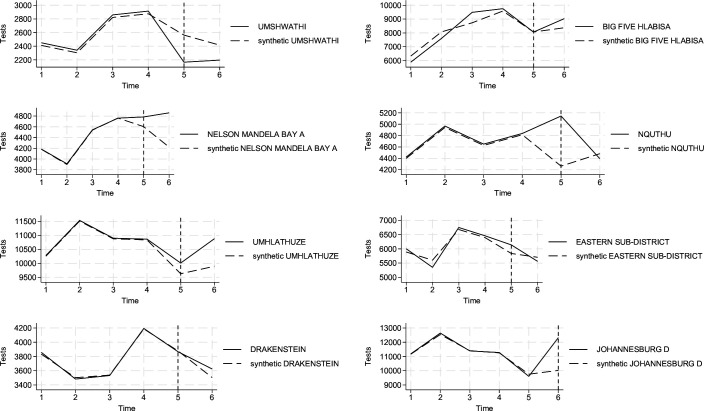
Heterogeneous impacts of intervention by subdistrict on TB tests per quarter, synthetic control models using quarterly NHLS data, period 2021 quarter 4–2023 quarter 1). Notes: the models include six time periods corresponding to quarters 2021q4 to 2023q1. Corresponding estimates and pseudo p-values are displayed in online supplemental table C5. NHLS, National Health Laboratory Service.

The results of the synthetic control analysis are presented in figure 3, showing trends for each treated subdistrict and its synthetic counterpart. Prior to activations, intervention-control unit trends matched well (details in online supplemental material C5). There were significant and positive effects for just two subdistricts. In the first activation quarter (ie, 2022 quarter 4 and 2023 quarter 1, respectively), Nquthu and Johannesburg D saw an increase of 880.19 (pseudo p-value=0.04) and 2300.04 (p-value=0.01), respectively. In the second quarter following the intervention, the effect for Nquthu turns negative and insignificant. In both cases, estimates far exceed the number of screeners advised to get a TB test via the platform (recall online supplemental material C2): 276 in Nquthu and 570 in Johannesburg D, suggesting that estimates captured confounding factors or that some intervention campaigns led to more TB testing without prior usage of the screening app.

### Spillovers

In some intervention subdistricts, a large increase in screens was observed in adjacent subdistricts (details in online supplemental material C6–C8). Figure 1 panels C and D re-estimated the dynamic difference-in-difference models on data aggregated at the district level to gauge possible spillovers from treated to untreated areas. We considered a district treated if one of its subdistricts was treated. We found suggestive evidence of spillovers as screens and positive screens increased by 655.33 (p-value<0.01) and 238.247 (p-value<0.01) respectively, and both estimates were qualitatively larger than the subdistrict ones in panels A and B. Excluding control subdistricts located in districts with a treated subdistrict in the fixed-effects models in table 2 did not qualitatively change the results (panels A and B, column 5).

## Discussion

We robustly documented that activation activities in subdistricts led to short-term increases in weekly TBCheck usage relative to control subdistricts as evidenced by the platform’s dashboard data. We also found that there were positive spillovers to neighbouring districts in the usage of TBCheck. Using NHLS data, we then tested if the increase in self-screenings translated to changes in weekly TB tests, positive tests, as well as the positivity rate. In line with the brief activity increases on TBCheck, we found a short-term effect on the number of tests but not on case findings nor the positivity rate. In other words, TBCheck promotion activities did not lead to sustained effects on TB testing beyond the initial week of activities nor on TB case detection.

Our study has limitations: (1) intervention subdistricts were not picked randomly for logistical and programmatic reasons. Our estimates, specifically in quarterly data, may thus be driven by dynamic confounders, including time-variant unobservables or other health programmes and shocks occurring at the same time, leading to violation of the parallel trends assumption. (2) Only a few heterogeneous subdistricts received intervention activities due to budget constraints. (3) Activation activities had low coverage and were of low and variable intensity, which may have contributed to the lack of sustained and large effects, and we may lack power to detect more subtle longer term effects. Effects were clearly concentrated in the short term and thus limited our ability to document heterogeneity and longer term effects using synthetic control models. Testing the effects of longer term interventions is a possible avenue for future research. (4) We presented evidence that activities spilled over to neighbouring areas, leading to benefit sharing. (5) We evaluate the impact of specific community activities and not the effects nor the specificity of the screening platform alone or mHealth in other contexts with different TB burdens and dynamics. (6) We lack information on intervention subdistricts that could further help to refine the analysis, including ‘missing TB cases’ and TB incidence. That said, such population trends are slow-moving, while we examine effects within weeks of a brief intervention. The subdistrict fixed effects absorb time-invariant confounders. (7) A final limitation of some of the intervention strategies is that individuals with active TB may feel unwell and may thus not be found in shopping centres. However, activities were therefore also covered in clinics.

## Conclusion

Community promotion activities briefly boosted the usage of a mHealth platform for TB self-screening and translated into brief increases in TB testing. These effects were not sustained, and there was no increase in positive case detection. Longer-term approaches and more sustained interventions and investments would be needed to yield enduring and large increases in self-screening and case detection at the population level. While mobile health screening tools have the potential to reach large shares of the population at risk, uptake of such tools remains a major challenge to solve for practitioners and policy stakeholders. To accelerate learning, we need better research and assessment of existing platforms and tools, and in particular assessments including the impact of screens on testing and case identification.

## Supplementary material

10.1136/bmjhci-2024-101179 online supplemental file 1

## Data Availability

Data may be obtained from a third party and are not publicly available.
