# Supplementary material for: Is it possible to encourage TB testing and detect missing TB cases via community-level promotion of a self-screening mobile application? Quasi-experimental evidence from South Africa
Source: BMJ Health Care Inform. 2025 May 31;32(1):e101179. doi: 10.1136/bmjhci-2024-101179 (PMC12128445; doi:10.1136/bmjhci-2024-101179)

**Supplemental Materials**

**Supplemental Material A:** Screening Questions TB Check and Testing Advice

1.Do you have a cough? Yes; No

2.Do you have a fever? (when you touch your forehead, does it feel hot?) Yes; No

3.Are you sweating more than usual at night? Yes; No

4.Have you been losing weight without trying? Yes; No

5.Are you at risk of TB? I am at high risk of TB; I am not at high risk of TB; I don’t know if I am at high risk

You are at risk of TB if:

*-You live with someone who has/had TB in the last year*

*-You have had TB in the last 2 years*

*-You are living with HIV.*

🡺Your replies to the questions show that you need a TB test this week

- Go to your local clinic for a free TB test.
- Please put on a face mask before you enter the clinic.

**Supplemental Material B:** Sub-District Selection

The intervention rollout was staggered to enable our research design and for logistical and feasibility reasons. Sub-districts (SDs) were selected as follows: The urban SDs were purposively selected by implementing partners with input from the funder. The rural SDs (uMshwathi, Big 5 Hlabisa and Nquthu) were picked from the target provinces Gauteng, KwaZulu-Natal and Western Cape. The sample was restricted to those rural SDs with more than 19 and less than 30 facilities based on information from the Department of Health's National Indicator Data Set; this range was deemed feasible for community interventions. From that list, three SDs were selected taking logistics and experience by the intervention implementing partner into account. After these three SDs were selected and before roll-out, the research team ran synthetic control models using national data to see if for these SDs one could find synthetic counterfactuals using historical TB testing data. For this exercise matching was done on 14 quarters (2018-2021 quarter 3), while the last quarter of 2021 was used for prediction and falsification tests. These falsification tests tested for impacts where there should be none as the intervention had not started. The three SDs passed these falsification tests and were thus chosen for the interventions.

**Supplemental Material C:** Supplemental Tables and Figures

Supplemental Material C1: Activation activities by date and sub-district

| **Sub-district (province)** | **Dates** | **Sites** | **Population 2021** | **Predominantly urban/rural** |
| --- | --- | --- | --- | --- |
| uMshwathi (KwaZulu-Natal) | 3 –8 October 2022 | Gcumisa Clinic  Wartburg (main road & Spar) | 101,720 | Rural |
| Big 5 Hlabisa (KwaZulu-Natal) | 10 –15 October 2022 | Hluhluwe Clinic  Hluhluwe taxi rank & Spar | 107,886 | Rural |
| Nelson Mandela Bay sub-district A (Eastern Cape) | 10 –15 October 2022 | Motherwell CHC  Motherwell Taxi rank & Boxer store  Motherwell Shopping Centre | 436,072 | Urban |
| Nquthu (KwaZulu-Natal) | 24 –29 October 2022 | Charles Johnson Memorial Clinic  Boxer store, Nquthu Plaza | 174,689 | Rural |
| uMhlatuze (KwaZulu-Natal) | 31 October–5 November 2022 | Nseleni Clinic  Empangeni Spar, taxi rank and surrounds | 459,646 | Urban |
| Cape Town Eastern health sub-district (Western Cape) | 21–25 November 2022 | Kleinvlei CDC  Blue Downs Shopping Centre | 726,150 | Urban |
| Drakenstein (Western Cape) | 28 November–3 December 2022 | TC Newman CDC  Rembrandt Mall  Paarl CBD  Shoprite Mbekweni | 314,023 | Urban |
| Johannesburg sub-district D (Gauteng) | 23–28 January 2023 | Chiawelo CHC  Dobsonville taxi rank  Protea Gardens mall | 1,529,355 | Urban |

Notes: Johannesburg sub-district D was activated relatively later. Accordingly, it only contributes impact estimates over a reduced intervention period in the dynamic difference-in-difference and fixed effect models, and only features one impact estimate in the synthetic control models.

Supplemental Material C2: Number of total screens and positive screens on TB Check in intervention sub-districts before and after interventions took place

|  | **Total screens** | | **Positive screens** | |
| --- | --- | --- | --- | --- |
| **Sub-district** | **Post-intervention**  (2022w40-2023w8) | **Pre-intervention**  (2022w1-2022w39) | **Post-intervention**  (2022w40-2023w8) | **Pre-intervention** (2022w1-2022w39) |
| UMSHWATHI | 70 | 5 | 35 | 1 |
| BIG FIVE HLABISA | 730 | 7 | 326 | 4 |
| NELSON MANDELA BAY A | 134 | 41 | 76 | 31 |
| NQUTHU | 939 | 22 | 276 | 3 |
| UMHLATHUZE | 875 | 54 | 287 | 31 |
| EASTERN SUB-DISTRICT | 629 | 250 | 218 | 102 |
| DRAKENSTEIN | 287 | 27 | 107 | 20 |
| JOHANNESBURG D | 1303 | 919 | 570 | 372 |
| **Total** | **4967** | **1325** | **1895** | **564** |

Note: Positive screens refer to screens resulting in the user being advised to test for TB.

Supplemental Material C3: TB Check Screens, raw weekly usage data 2022w1-2023w8


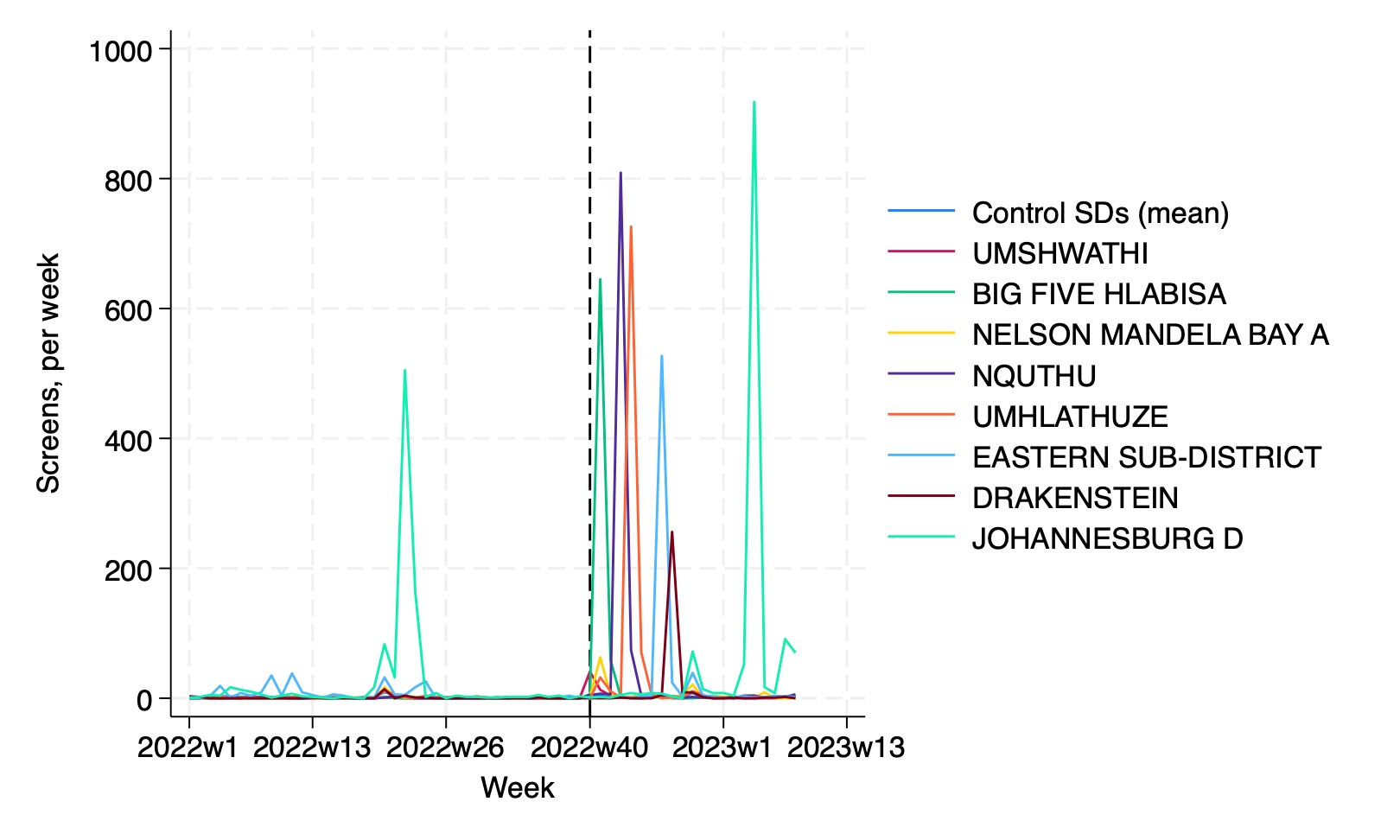


Note: The dashed line shows the date of the start of the first intervention. Other interventions took place over the subsequent weeks, corresponding with the spikes in each sub-district.

Supplemental Material C4: Average impact on number of tests in log form, weekly NHLS data 2022 Week 1–2023 Week 8

|  | **(1)** | **(2)** | **(3)** | **(4)** | **(5)** |
| --- | --- | --- | --- | --- | --- |
| Impact | 1.26*** | 1.35*** | -0.08* | 0.003 | 0.003 |
|  | (0.20) | (0.21) | (0.05) | (0.05) | (0.05) |
| Week FEs |  | x |  | x | x |
| Sub-district FEs |  |  | x | x | x |
| R^2^ | 0.01 | 0.04 | 0.88 | 0.90 | 0.90 |
| N | 14760 | 14760 | 14760 | 14760 | 12660 |

Notes: FE denotes fixed effect (exploiting within sub-district and week variation). 'x' indicates whether week and/or sub-district fixed effects were included. Column 1 includes no fixed effects, while columns 4 (preferred model) and 5 include both week and sub-district fixed effects. Standard errors in parentheses are clustered at the sub-district level. Column 5 excludes sub-districts in districts with treated sub-districts to account for possible spillovers. *** p<0.01, ** p<0.05, * p<0.1

Supplemental Material C5: Synthetic control estimates of impacts on TB tests per quarter, NHLS data (period 2021 quarter 4–2023 quarter 1)

| **Sub-district** | **Impact 2022q4**  **(pseudo p-values)** | **Impact 2023q1**  **(pseudo p-values)** |
| --- | --- | --- |
| UMSHWATHI | -396.75 (0.16) | -219.85 (0.38) |
| BIG FIVE HLABISA | -6.27 (0.97) | 658.27 (0.14) |
| NELSON MANDELA BAY A | 179.72 (0.37) | 638.71 (0.16) |
| NQUTHU | 880.19 (0.04) | -86.34 (0.66) |
| UMHLATHUZE | 382.55 (0.17) | 986.02 (0.08) |
| EASTERN SUBDISTRICT | 302.10 (0.23) | -143.55 (0.47) |
| DRAKENSTEIN | -11.53 (0.95) | 119.77 (0.53) |
| JOHANNESBURG D | Not activated yet | 2300.04 (0.01) |

Supplemental Material C6: TB Check Screens in uMshwathi District and adjacent sub-districts, raw weekly dashboard data 2022w1-2023w8


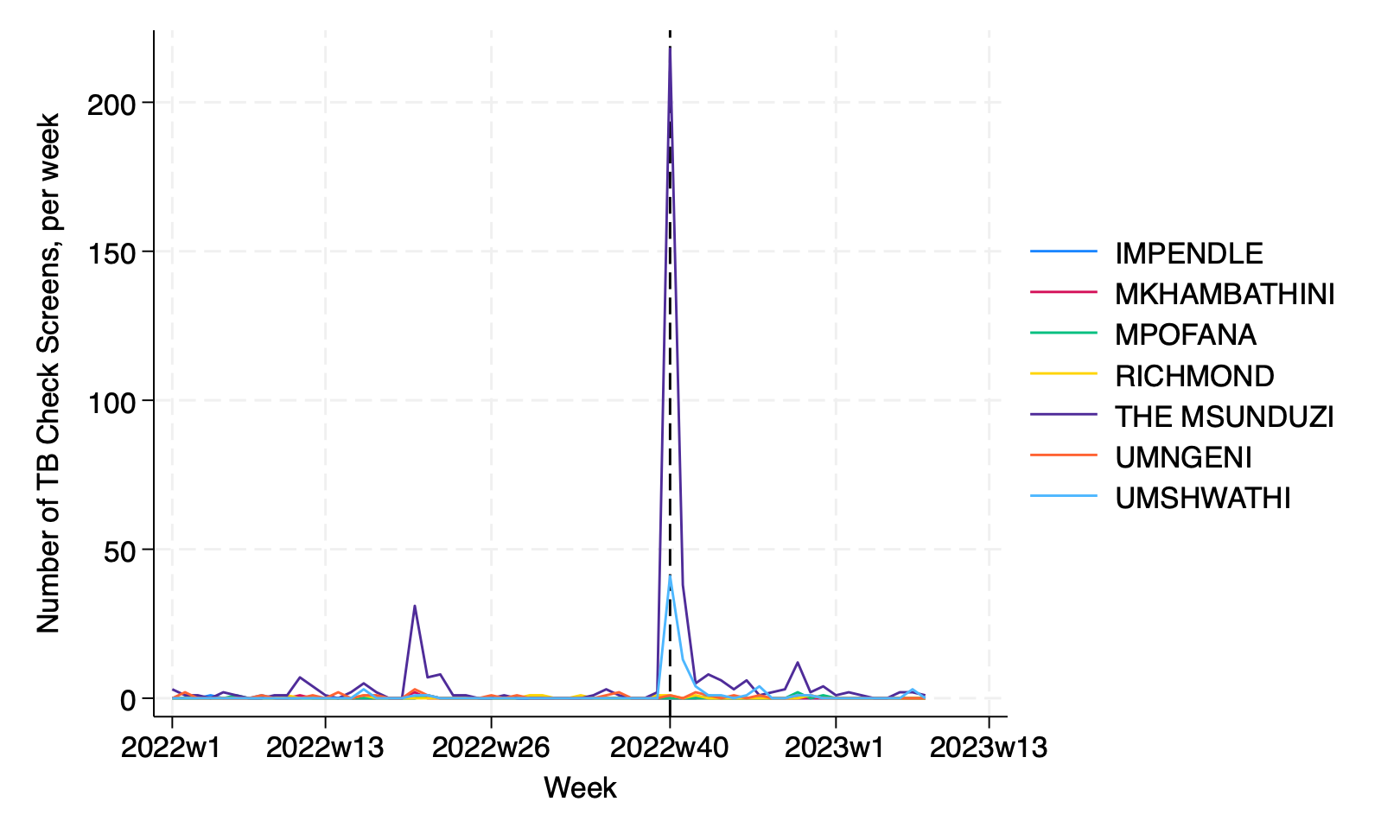


Supplemental Material C7: TB Check Screens in Nelson Mandela Bay A and adjacent sub-districts, raw weekly dashboard data 2022w1-2023w8


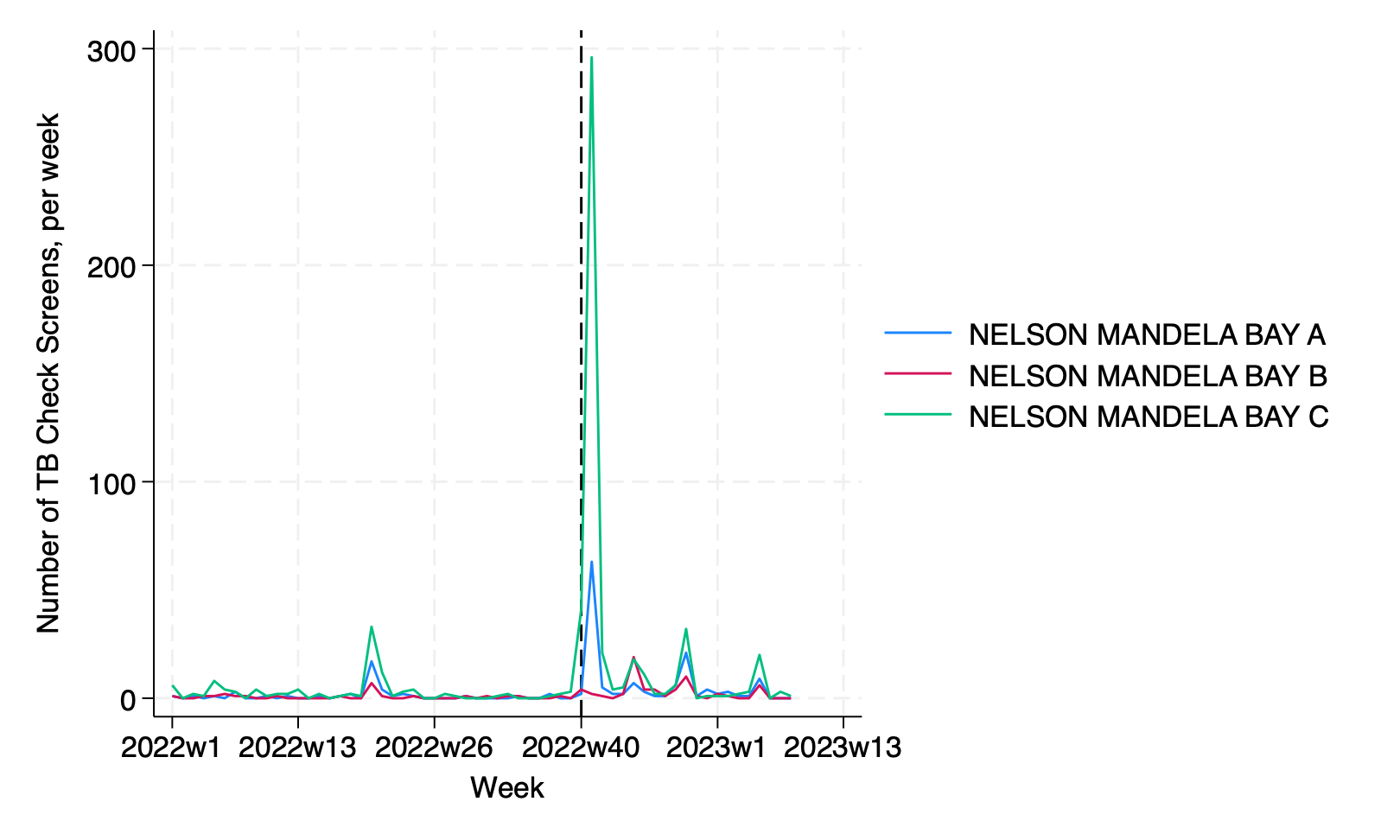


Supplemental Material C8: TB Check Screens in Johannesburg D and adjacent sub-districts, raw weekly dashboard data 2022w1-2023w8


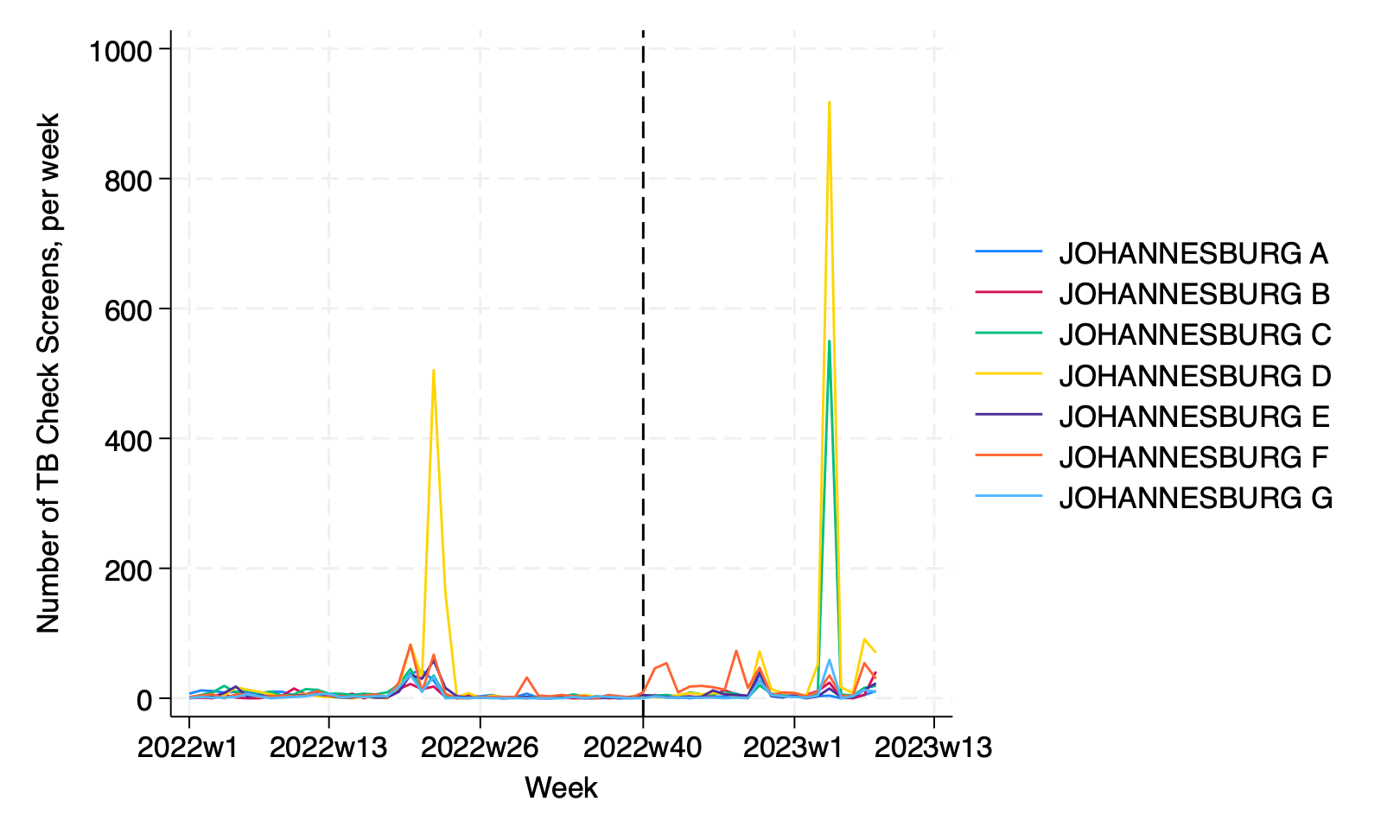

Supplement: online supplemental file 1 [file bmjhci-32-1-s001.docx]
